# Performance of Reinforced Concrete Beams Strengthened with Carbon Fiber Reinforced Polymer Strips

**DOI:** 10.3390/ma14195866

**Published:** 2021-10-07

**Authors:** Muhammad Haroon, Jae Sang Moon, Changhyuk Kim

**Affiliations:** 1 Department of Architectural Engineering, Sungkyunkwan University, Suwon 16419, Korea; haroon12@g.skku.edu; 2 Department of Civil Engineering, Balochistan University of Engineering and Technology, Khuzdar 89100, Pakistan; 3 Structural Department, Yooshin Engineering Corporation, Seoul 06252, Korea; mjaesang@gmail.com; 4 Department of Architectural Engineering, Inha University, Incheon 22212, Korea

**Keywords:** CFRP, shear strengthening, unidirectional layout, bidirectional layout

## Abstract

Carbon fiber reinforced polymers (CFRP) have shown considerable potential in the repair and rehabilitation of deficient reinforced concrete (RC) structures. To date, several CFRP strengthening schemes have been studied and employed practically. In particular, strengthening of shear damaged RC members with CFRP materials has received much attention as an effective repair and strengthening approach. Most existing studies on strengthening shear-deficient RC members have used unidirectional CFRP strips. Recent studies on strengthened T-beams demonstrated that a bidirectional CFRP layout was more effective than a unidirectional layout. As such studies are limited, in this study, the feasibility of bidirectional CFRP layouts for the shear strengthening of rectangular RC beams was experimentally evaluated. Bidirectional layout details with CFRP anchors as well as rehabilitation timing were considered and investigated. The test results showed that the members with a bidirectional CFRP layout carried less shear strength capacity than those with unidirectional layouts for the same quantity of CFRP material. Nevertheless, the bidirectional CFRP layout allowed for a uniformly distributed stirrup strain compared to the unidirectional CFRP layout at the same load level, which increased the efficiency of the transverse reinforcement. Additionally, the shear contribution of CFRP material according to the CFRP strengthening timing was verified.

## 1. Introduction

Aging reinforced concrete (RC) structures undergo strength deterioration owing to extreme environmental and loading effects. Effective retrofitting techniques can enable structural members to regain structural capacities equal to or even higher than the designed capacities in a cost-effective manner. To date, several conventional and advanced strengthening techniques, such as section enlargement, external prestressing, and steel plate bonding and materials such as externally bonded fiber-reinforced polymers (FRP) have been effectively implemented for the rehabilitation of RC structures. In the last few decades, externally bonded carbon FRP (CFRP) materials have been widely used for such applications owing to their flexible usability, cost-effectiveness, high strength-to-weight ratio, high corrosion resistance, low thermal conductivity, and improved structural performance under critical loading conditions. In particular, strengthening the shear deficient members with CFRP is an efficient technique for upgrading the shear capacity [1]. Experimental studies on the shear behavior of strengthened RC beams with CFRP focused on several important parameters such as wrapping schemes and layouts, the angle of inclination of CFRP strips, the quantity of CFRP material and anchorage, and the effective number of CFRP layers. For instance, Norris et al. [2], Thanasis [3], Islam et al. [4], and Zhang [5] investigated the effect of CFRP strip orientation and demonstrated that the effectiveness of CFRP increases as the strip direction becomes nearly perpendicular to the shear crack direction. Furthermore, the effectiveness of bonded CFRP materials in restricting the diagonal crack widths was dependent on the amount of reinforcement, orientation, and bond characteristics. Adhikary and Mutsuyoshi [6] reported that increasing the number of layers and depth of carbon fiber sheets increased the shear strength, and among the various wrapping schemes, the vertical U-wrap of the sheet provided the most efficient strengthening. Similarly, several studies [1,7,8,9,10,11,12,13,14,15,16] investigated the behavior of full-scale T-beams strengthened in shear with CFRP, reporting that the effectiveness of CFRP strengthening on shear resistance was influenced by the quantity of shear reinforcement used. Denuiaud et al. [13] observed that the composites were less effective for shear in heavily reinforced beams. Although the plane sections did not remain plane in the shear span after a certain load level, the external FRP sheets delayed the loss of the plane section behavior. The shear contribution by the arch action was also delayed owing to CFRP. At ultimate loads, the remaining beam action accounted for approximately 20% of the total shear force when the members were reinforced with a significant quantity of shear reinforcement, either with the conventional stirrups or externally bonded CFRP sheets. In general, the maximum shear increased with an increasing number of CFRP layers; however, increasing the shear did not depend on the number of CFRP layers alone. The optimum number of layers to achieve the maximum gain in shear resistance was found to depend on the amount of internal shear reinforcement [9]. The beam strengthened with CFRP strips only on the sides contributed less shear strength compared to that contributed by the U-wrap specimens [7]. Taljsten [17] agreed that the shear strengthening of RC beams becomes more effective when CFRP laminates are placed perpendicular to the crack direction. The structural members could easily be over strengthened; however, the compressive strength of concrete limits the shear strengthening. In an experimental study on the debonding failure state, Cao et al. [18] investigated the distribution of strains in CFRP strips that intersect the critical shear crack and the shear capacity at debonding. Pellegrino and Modena [19] tested shear-strengthened rectangular RC beams, reporting that the interaction mechanism between the externally bonded CFRP and the internal shear reinforcement strongly influenced the efficiency of the shear strengthening, which is ignored in present-day design codes. Sarah et al. [20] demonstrated that, by providing numerous small anchors with a total cross-sectional area at least two times greater than that of the longitudinal sheet, it was possible to induce fracture failure in CFRP sheets. Kim et al. [21] showed that the variable shear span-depth ratios (*a/d*) influenced the failure mode of the shear strengthened members; therefore, it is recommended to consider the design of CFRP strengthening schemes. Chalioris et al. [22] tested five shear-critical RC beams retrofitted by U-shaped jackets made of cementitious mortar and reinforced with small-diameter mild steel bars and U-shaped open stirrups. The test results showed that the shear strength of the retrofitted beams was substantially increased. The increase of the shear stress ranged from 38 to 48%. Chalioris et al. [23] investigated the effectiveness of U-jacketing in shear-critical through externally bonded FRP as shear reinforcement. They indicated that although the CFRP strengthened beams exhibited increased shear capacity, the brittle failure mode could not be prevented due to the debonding. Nevertheless, the deboning of CFRP sheets was delayed due applied mechanical.

Although most of the studies discussed above used unidirectional CFRP strips to strengthen shear-deficient RC members, some [14,24,25,26] utilized bidirectional CFRP strips to strengthen the RC I-girders and panels. These studies demonstrated that the use of bidirectional CFRP is more efficient than that of unidirectional CFRP. Kim et al. [14] reported the test results for four shear-strengthened I-girders, which compared the performance of uni- and bidirectional CFRP with a control specimen. The results showed that the use of anchored unidirectional CFRP could increase the shear capacity slightly (2%), while a significant increase (up to 40%) in shear capacity was observed in members with bidirectional CFRP strips. Although these results indicated that the performance of the bidirectional application of CFRP was superior, the mechanism that resulted in the improved performance remains unclear. Alotaibi [25] tested eight shear-strengthened RC T-beam sections similar to typical bridge girders with anchored bidirectional CFRP strips to investigate their behavior and make a direct comparison with the results reported by Kim et al. [14]. The test results showed that the efficiency of the bidirectional CRFP strips was significantly influenced by the shear span-depth ratio (*a/d*). For members with short *a/d*, the effect was negligible, while for members with *a/d* equal to 3, the bidirectional CFRP increased the shear strength by up to 62% compared to that of the control specimens. The concrete and CFRP shear contributions showed an interaction, whereas the contribution of the internal shear reinforcement was found to be identical to its contribution in the case of non-strengthened RC beams. These tests were the first of their kind and provided very useful technical information on the behavior of bidirectional CFRP strengthened beams under shear. Nevertheless, these tests are limited in number and were carried out on the T-sections, and the results may not necessarily be true for members with rectangular sections. However, these observations can be used as a basis for further research to better understand the response of members strengthened with bidirectional CFRP for shear. Later, Kim et al. [24,26] tested bidirectional-CFRP-strengthened RC panels under compression loads to simulate the bottle-shaped compression strut mechanism developed in the CFRP-strengthened web elements of deep RC beams. The panel tests showed that the application of bidirectional CFRP strips significantly increased the cracking (30%) and maximum (150%) loads compared to the strengthened panels. Despite the relevance of RC panel tests, such observations cannot be applied directly to the beams because of the differences in the loading and boundary conditions. 

In this study, a testing program was planned and executed to investigate the behavior of rectangular RC beams strengthened with bidirectional CFRPs. Several studies have been conducted to investigate the shear behavior of strengthened RC beams with unidirectional CFRP strips and sheets. However, experimental studies on the use of bidirectional CFRP strips for this purpose are few. Therefore, in this study, a detailed experimental program was planned and executed to evaluate the potential use and effectiveness of bidirectional CFRP strips for strengthening rectangular RC beams under shear. A total of 18 RC beams were constructed and tested to evaluate various CFRP strengthening features such as the strengthening timing, presence of CFRP anchors, CFRP layouts, etc. 

## 2. Experimental Program

### 2.1. Test Parameters and Specimen Details

The experimental program consisted of two series of tests. In the first series, a total of ten RC beams were tested, while in the second series, eight specimens were tested under shear. The main variables studied were the effect of shear stirrup spacing, amount, layout, and configurations of CFRP strips, and the effect of pre-cracking on repair and strengthening. 

**Figure 1:**
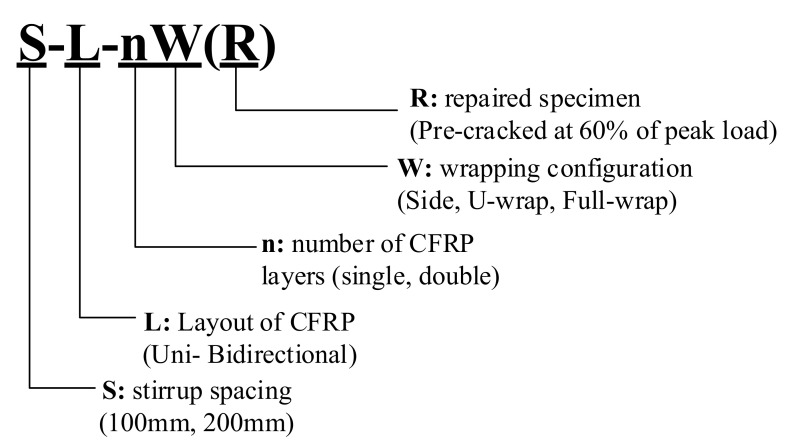
Nomenclature of test specimens.

Figure 1 shows the nomenclature used in this study. The nomenclature of the test specimens has either four or five characters. The first character “S” indicates the transverse reinforcement spacing, which was either 100 or 200 mm. The second term “L” represents the layout of the CFRP strips, where C stands for the control specimen (non-strengthened), U indicates the unidirectional layout (transverse direction only), and B represents the bidirectional layout of the CFRP strip. The third term “n” indicates the number of layers of CFRP used, which can be either 1 or 2. The term “W” stands for wrapping configurations where “F,” “U,” and “S” were used for fully wrapped, U-wrapped, and side-bonded configurations, respectively. Lastly, “R” indicates that the specimen was repaired after pre-loading for up to 60% of the designed loads and subsequently, unloaded, repaired, and reloaded up to failure.

Table 1 shows the details of the specimen cross-sections and CFRP strips. The RC beams in both the test series had a cross-sectional dimension of 300 mm × 500 mm, but the length of the specimens in the first series was 2600 mm, whereas the specimens in the second series had a length of 2200 mm. The specimens in the first and second series had shear span-to-depth ratios (*a/d*) of 3 and 2.1, respectively. The intended failure mode of the tested members was shear; therefore, the beams were designed to have a higher flexural capacity using the D25 longitudinal reinforcing bars. In the first series of tests, the entire length of the beams was considered as the test region, with shear span lengths of 1300 mm on either side of the concentrated loading point at the mid-span. Therefore, both shear spans were designed with similar sectional strengths and CFRP strengthening schemes. In the case of specimens in the second series, a span length of 870 mm between the left support and the loading point was taken as the test region while maintaining the high shear strength of the span between the loading point and the right support to avoid any cracking and failure. The right shear span of the members in the second series was designed using D13 reinforcing bars as shear reinforcement with a spacing of 100 mm compared to the left shear span with D6 rebars at 200 mm, which was the test region.

The parameters considered in the first series of tests were the spacing of shear stirrups, the uni- and bidirectional layouts of the CFRP strips, and the effect of pre-cracking on repair and strengthening. The fully wrapped configuration of the vertical CFRP strips was used in the first test series, whereas the horizontal CFRP strips were anchored with CFRP anchors. 200-C-00 and 100-C-00 were used as control specimens without CFRP strengthening and with stirrup spacings of 200 mm and 100 mm, respectively. Specimens 200-U-1F, 200-B-1F, 100-U-1F, and 100-B-1F had stirrup spacings of 200 mm or 100 mm, and one layer of fully wrapped vertical CFRP configuration with/without horizontal CFRP strips. 200-U-1F(R), 200-B-1F(R), 100-B-1F(R), and 100-B-1F(R) were repaired specimens obtained after pre-loading to 60% of the designed capacity. In the members with the bidirectional CFRP layout, the amount of CFRP was equally distributed in both the vertical and horizontal directions, while keeping the total amount the same as in members with unidirectional configuration. On the other hand, the main parameters in the second series of specimens were the CFRP layout and configuration. The specimens had either uni- or bidirectional layouts with fully wrapped, U-wrapped, and side-bonded configurations of CFRP strips. Both the uni- and bidirectional strengthened specimens had equal quantities of vertical CFRP strips. Additional CFRP material was used as the horizontal strip in the bidirectionally strengthened members. The shear stirrup spacing was maintained at 200 mm for the test regions of all the beams tested in the second series. Specimens 200-U-1S, -1U, -1F and 200-B-1U, -1S, -1F were designed with uni- or bidirectional CFRP layouts, but with side-bonded, U-wrapped, and fully wrapped configurations, respectively. Finally, specimen 200-U-2F had double layers of U-wrapped unidirectional CFRP strips.

After RC beam fabrication, the concrete surfaces of the test specimens were ground and smoothened using a concrete grinder so that the CFRP strips could be bonded effectively. Anchor holes were also processed for CFRP anchor application. For the anchor installation, the holes were drilled with an embedment length of 100 mm and a diameter of 15 mm. As per ACI440.2R-08 [27] recommendations, all the 90-degree sectional edges were rounded to have a 13 mm radius chamfer in order to minimize the stress concentration at the corners. The CFRP strips were bonded to the shear area after the epoxy primer was used. In the first series of tests, the vertical CFRP strips overlapped, and the horizontal strips were held using anchors to prevent CFRP delamination failure. The ratio of the anchor area to the strip area was equal to two for the CFRP anchor fabrication. The application process of the CFRP strip is also described in Kim et al. [14]. Details of the CFRP strips are shown in Table 1. In the case of unidirectionally strengthened specimens, the width of the vertical CFRP strips was chosen to have a nearly identical shear contribution to that of the transverse reinforcement. In the second series, the vertical CFRP strips in the side-bonded and U-wrapped configurations were simply bonded with epoxy resin without anchors, assuming additional support from the horizontal CFRP strips.

**Figure 2:**
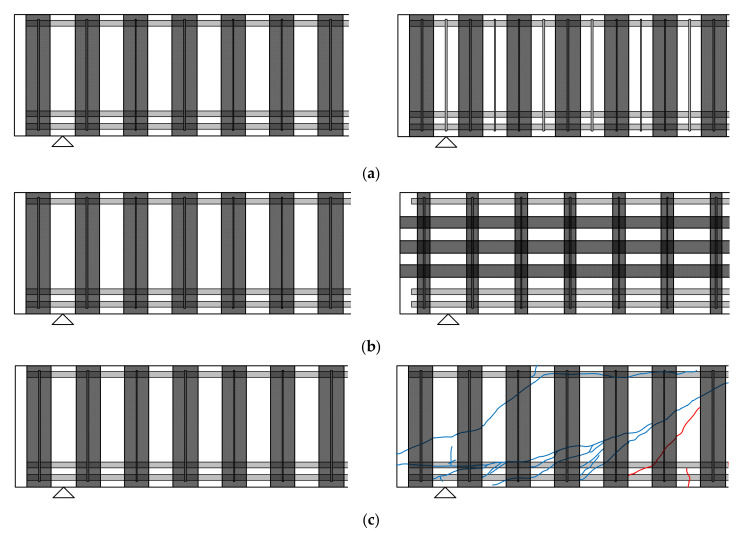
Main parameters of test specimens in the first series: (**a**) 200 mm vs. 100 mm stirrup spacing; (**b**) uni-directional vs. bi-directional CFRP layout; (**c**) uncracked vs. pre-cracked.

**Figure 3:**
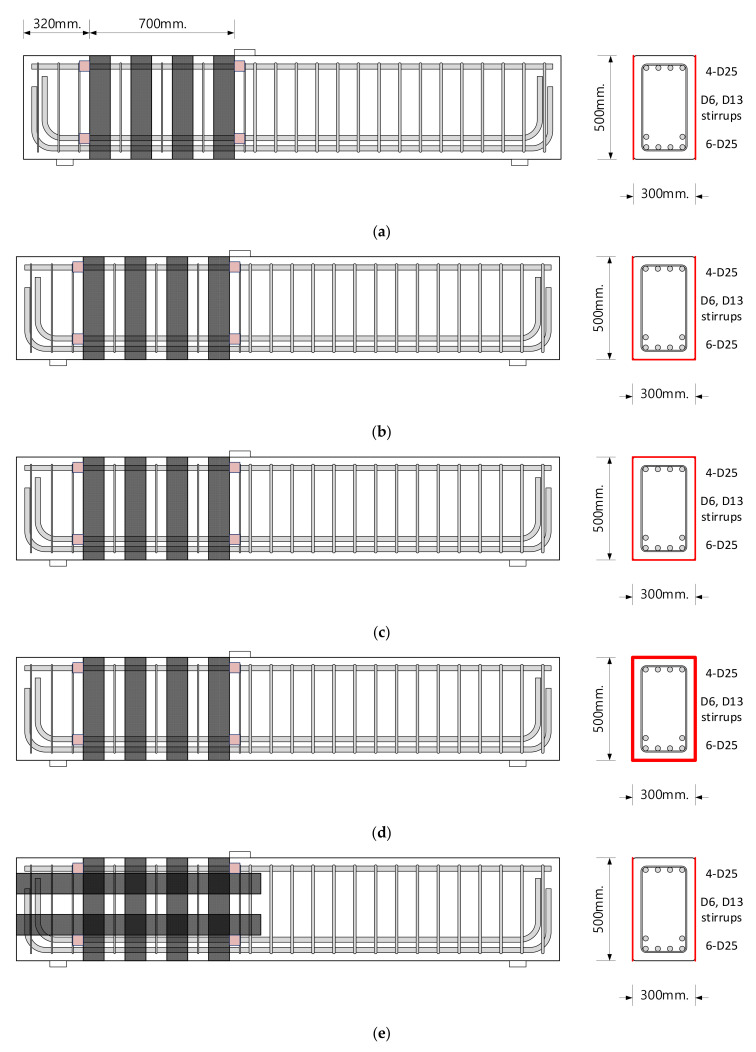

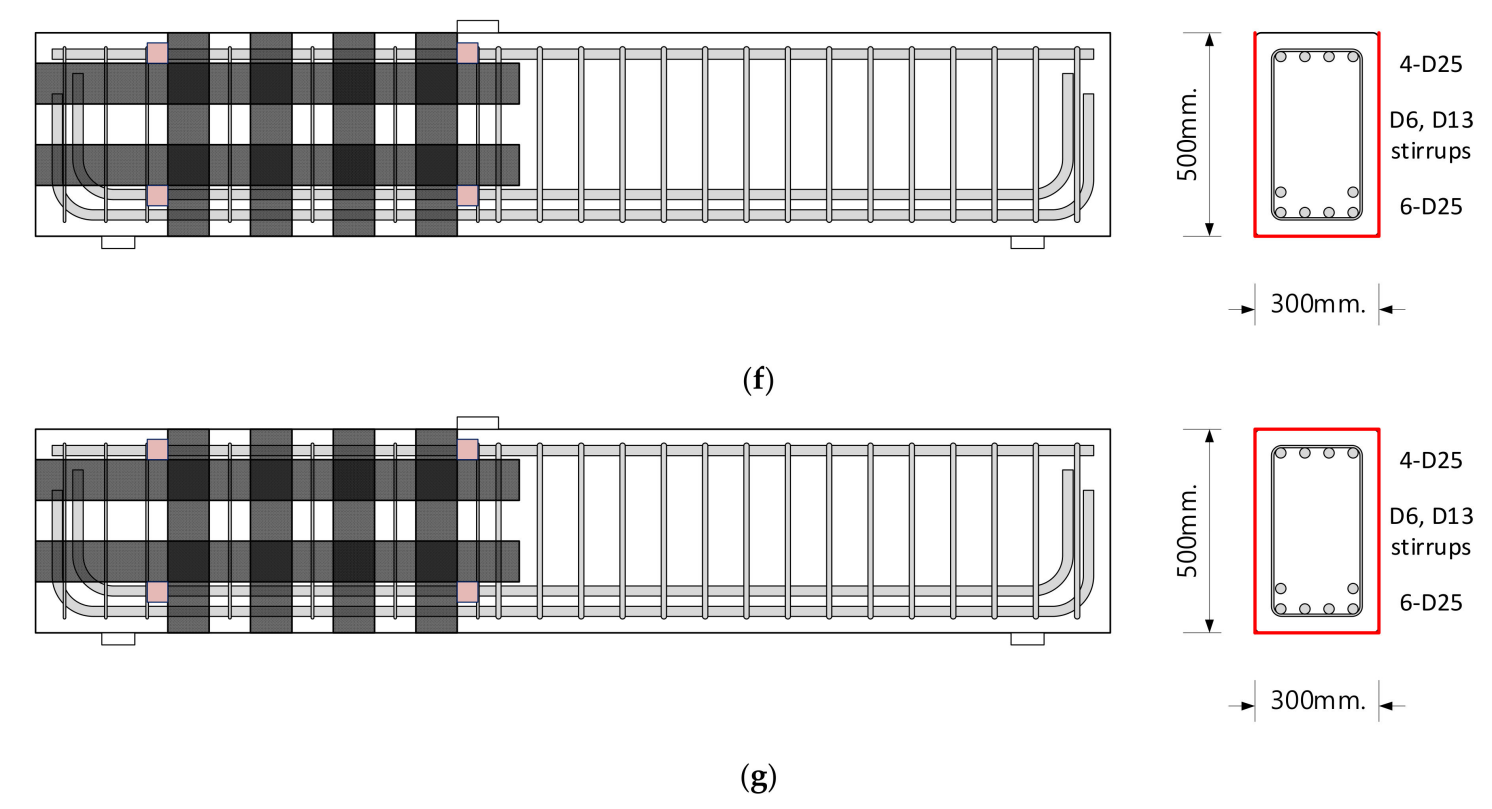
Main parameters of test specimens in the second series: (**a**) unidirectional side-bonded CFRP; (**b**) unidirectional U-wrapped CFRP; (**c**) unidirectional fully wrapped CFRP; (**d**) unidirectional fully wrapped with two layers of CFRP; (**e**) bidirectional side-bonded CFRP; (**f**) bidirectional U-wrapped CFRP; (**g**) bidirectional fully wrapped CFRP.

Figure 2 and Figure 3 represent the CFRP layout, configurations, and other variables considered in the first and second series of tests, respectively. As shown in Figure 2a, the specimens were designed to have different stirrup spacings with identical CFRP layouts. A stirrup spacing of 200 mm and 100 mm was applied to the first series of beams. The shear contribution of each reinforcing material was evaluated based on this group of specimens. Figure 2b shows different CFRP layouts with identical quantities of CFRP in the shear span. In the unidirectional CFRP strengthened beams, the vertical CFRP strip width and spacing were 100 mm and 200 mm, respectively. The vertical CFRP strip width was 50 mm for the bidirectional CFRP layouts. The width of the vertical strips in the bidirectional layout was half that of the unidirectional layout. However, an identical amount of CFRP strips was applied in the horizontal direction in the bidirectional layout. The effect of the CFRP layout with identical material amounts can be verified based on this series of specimens. Figure 2c represents two specimens to study the effects of pre-loading and repairing under a unidirectional CFRP layout. The specimen on the left side was strengthened with CFRP strips without pre-loading, while the specimen on the right side was pre-loaded to the service load level without CFRP strengthening, unloaded, repaired with CFRP strips, and reloaded to failure.

Figure 3 shows the specimens in the second series of tests, in which the CFRP layout and configuration were the variables. Figure 3a–d represent the side-bound, U-wrapped, and fully wrapped configuration of the CFRP strips in a unidirectional layout, while these configurations are shown in Figure 3e–g for the bidirectional layout. As discussed above, the quantity of vertical CFRP material was kept the same in both uni- and bidirectionally strengthened members.

### 2.2. Test Measurements and Setup

**Figure 4:**
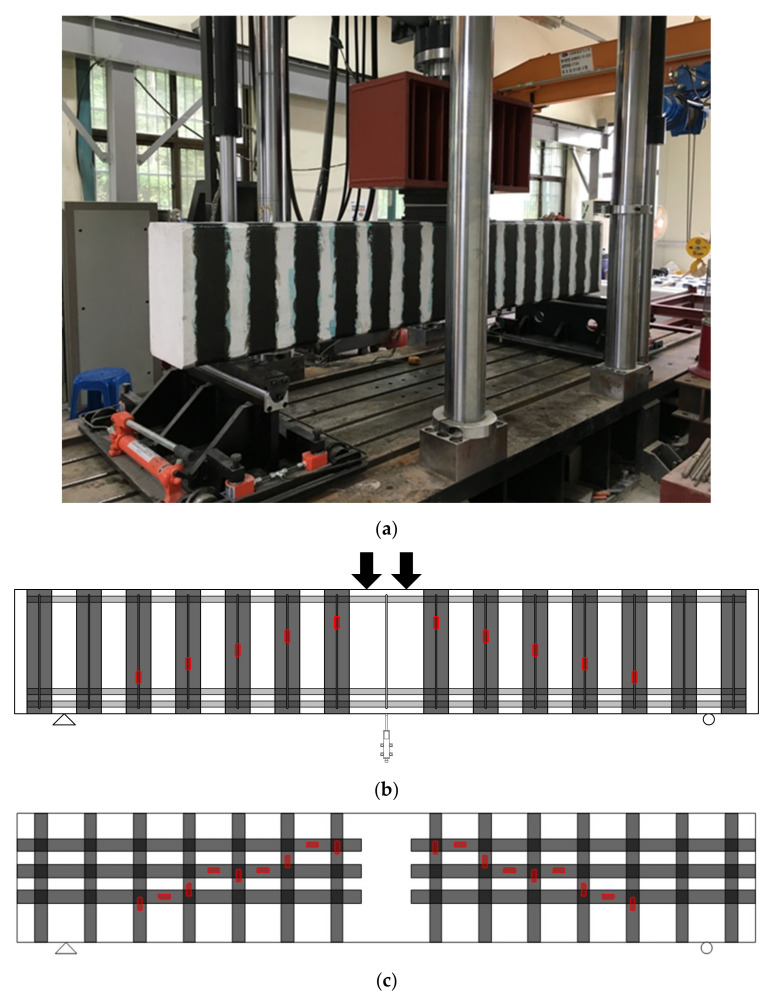
Test setup and schematic representation of LVDTs and strain gauge locations: (**a**) test setup, (**b**) LVDTs and strain gauges in members with unidirectional layout, and (**c**) LVDTs and strain gauges in members with bidirectional layout.

The specimens were tested under shear using a concentrated load transferred through a steel plate between two simple supports using a universal testing machine (UTM). Figure 4 shows the test setup, location of linear voltage displacement transducers (LVDTs), and strain gauges on the CFRP strips. Two LVDTs were installed to monitor the deflection of the specimens at the loading point. Strains of the transverse reinforcement were measured using strain gauges attached to the stirrups. For the first series, in specimens with a unidirectional layout, a total of 10 strain gauges, while in the case of bidirectionally strengthened members, 18 strain gauges were attached along the expected diagonal cracks. The strain gauges for the stirrups were attached to an identical location as the CFRP gauges. The transverse reinforcement strain in the second series of tests was measured by attaching five strain gauges to the stirrups in the test region. The strain in the longitudinal flexural reinforcement was also measured using strain gauges attached to the loading points. The test specimens were loaded under a monotonic loading protocol until the beams reached 85% of the maximum load in the post-peak loading range. The load was applied to the beams using a deflection control rate of 0.01 mm/sec. In the case of repaired specimens, the beams were unloaded after reaching the service load level, which was taken as 60% of the designed capacity. The shear strengthening work was conducted using CFRP strips, and then the specimens were reloaded to failure.

### 2.3. Material Properties

The measured average compressive strength (*f*_c_^’^) of the cylindrical concrete specimen on the 28th day was 36.8 MPa and 31.8 MPa for the two series, respectively. Table 2 lists the material properties of the reinforcement used for the shear and flexure used in this study. The yield strengths (*f*_y_) of the flexural reinforcement were 661 MPa and 400 MPa, whereas the D6 steel bars used as shear reinforcement had *f*_y_ values of 341 MPa and 389 MPa in the first series of tests, respectively. The material properties listed in Table 2 were used to calculate the shear contribution of the transverse reinforcement and flexural capacity. The CFRP material test results were conducted according to ASTM D 3039, and the tensile strength and elastic modulus were 4600 MPa and 288,900 MPa, respectively.

## 3. Test Results 

### 3.1. Cracking and Failure Mode

**Figure 5:**
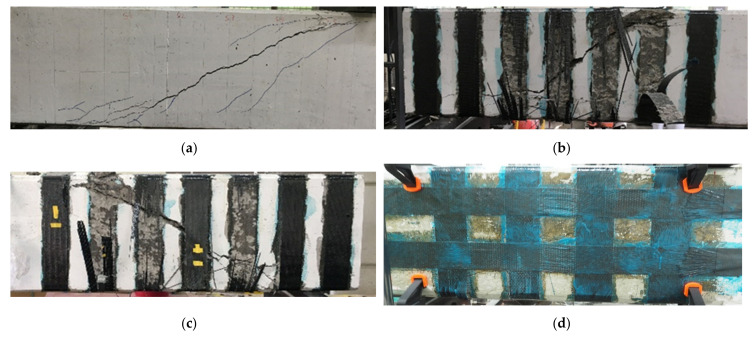

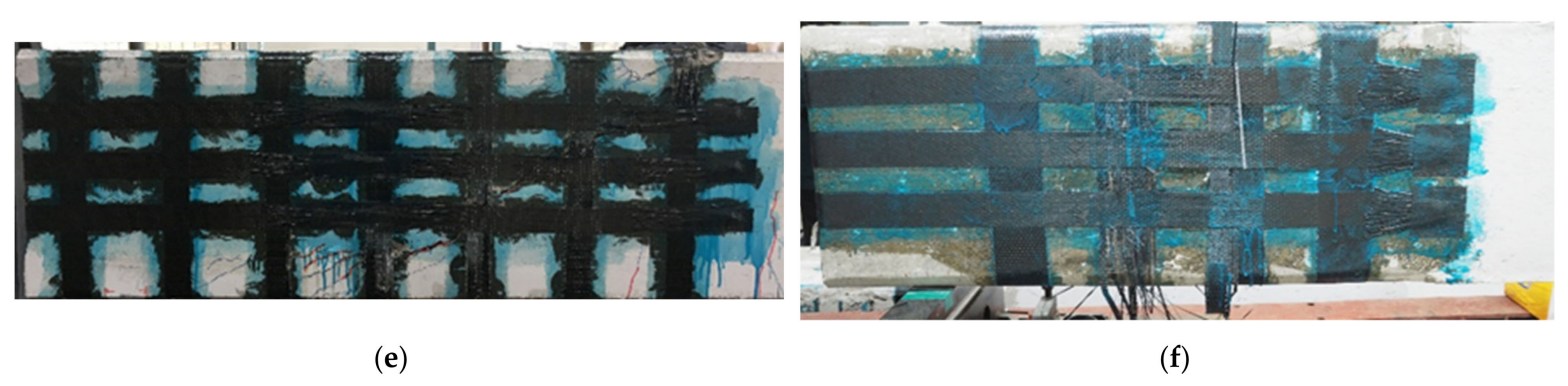
Cracking pattern and CFRP strip fracture in the representative specimens: (**a**) cracking and shear failure in control specimen (100-C-00); (**b**) CFRP fracture in fully-wrapped specimen with unidirectional layout (200-U-1F); (**c**) strip fracture in fully-wrapped repaired specimen with unidirectional layout (100-U-1F(R)); (**d**) CFRP fracture in side-bonded specimen with bidirectional layout (200-B-1S); (**e**) CFRP fracture in fully-wrapped repaired specimen with bidirectional layout (200-B-1F(R)); (**f**) CFRP fracture in fully-wrapped specimen with bidirectional layout (200-B-1F).

Overall, in the absence of flexural reinforcement yielding, the observed failure mode of the test specimens was shear, as intended in the design. Figure 5 shows the cracking pattern and CFRP strip fracture in the representative test specimens from the two series. In the control specimens, the cracks were first initiated in the bottom fiber under tension, which soon turned into inclined shear cracks that propagated from the support to the loading point. With a further increase in the load, a single major crack appeared in the web region whose width increased until the maximum load was reached. The cracking and failure patterns of the control specimens showed a clear shear-compression failure. A similar cracking mechanism was observed in the members strengthened using CFRP strips. Despite being shear-dominated, the CFRP strips delayed the failure and increased both maximum load and deflection. In almost all the members, the full strength of the CFRP material was successfully utilized. Around the ultimate load level, CFRP strip fractures were observed within the shear span of the test specimens. As the CFRP strips overlapped with a sufficient length in the first series, no delamination failure occurred. In the second series, the horizontal anchored strips provided additional support to the vertical CFRP strips in members with side-bonded and U-wrapped configurations; therefore, no CFRP debonding or delamination was observed. Table 3 shows the test results of the 18 specimens along with the observed failure modes. The shear capacities calculated using the ACI318-19 building code and the measured maximum shear capacity are listed in the table. The ratio of the test result to the design capacity of the specimens, the maximum strain of the stirrups, and CFRP strips are also presented in the table. The results show that in all the specimens, the stirrup reached the yield strain and the strengthened members failed owing to the CFRP strip fracture.

### 3.2. Load vs. Deflection Behavior

**Figure 6:**
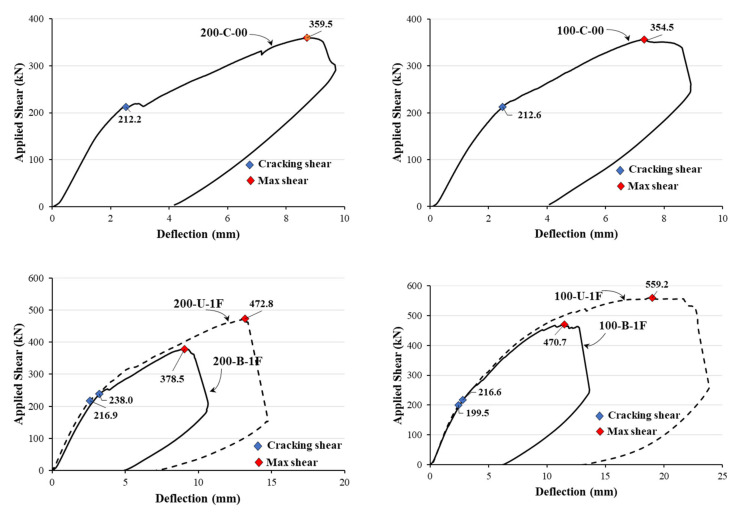

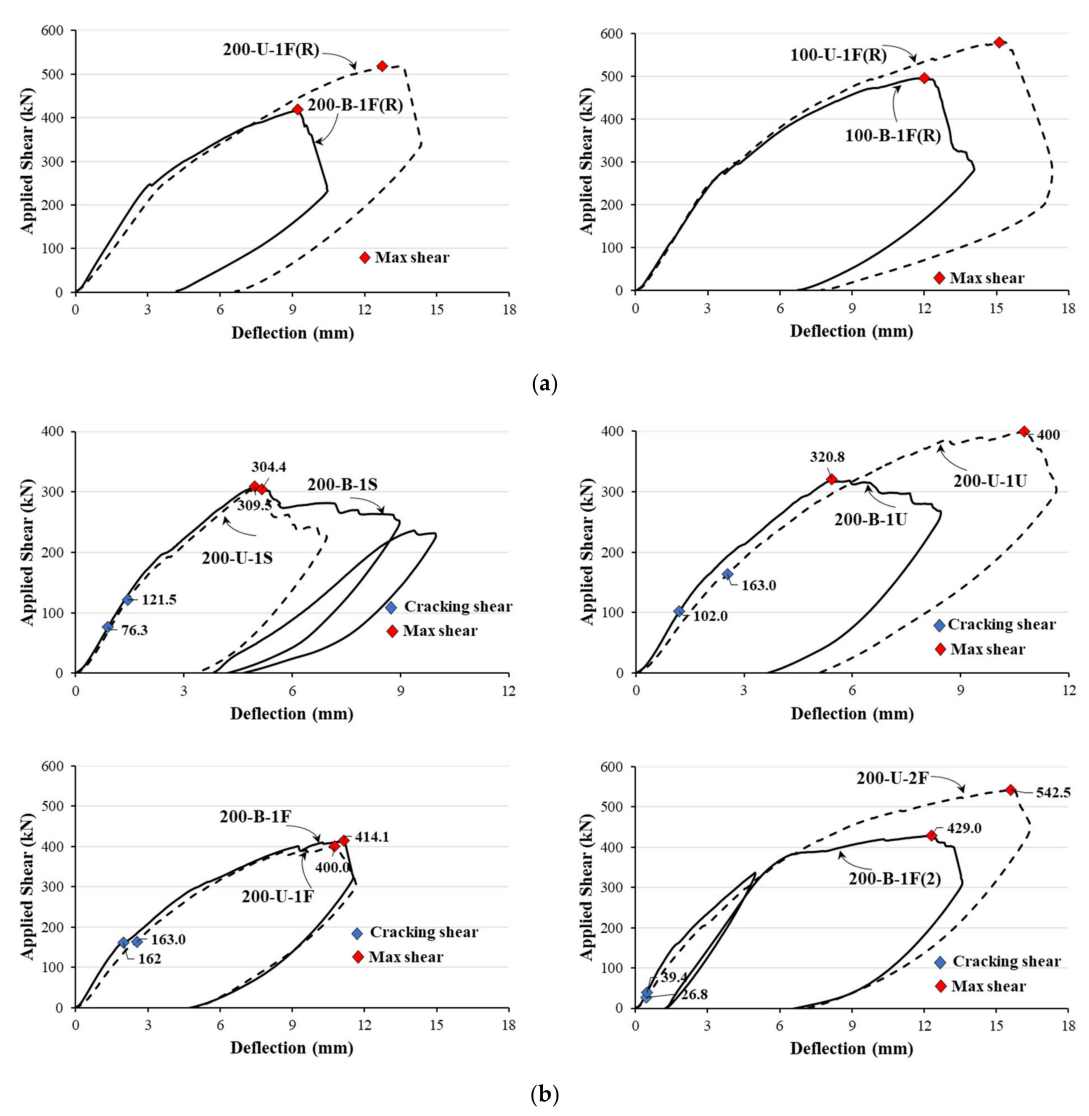
Load vs. deflection behavior of test specimens: (**a**) first series of specimens; (**b**) second series of specimens.

The measured load versus deflection behavior of the two series of tests is shown in Figure 6. The test results compare the effect of the CFRP layout (uni- and bidirectional) in specimens with the same stirrup spacing and CFRP configuration (side-bonded, U-wrapped, and fully wrapped). Table 3 presents the calculated shear force (*V*_ACI_) through ACI318-19 and the measured maximum shear (*V*_test_), stirrup strain (*ε*_s,max_), CFRP strain (*ε*_f,max_), and observed failure mode. The test results show that the measured maximum shear (*V*_test_) of the two control specimens, 100-C-00 and 200-C-00, was almost the same regardless of the stirrup spacing. This is because the failure mode of the two specimens was controlled by concrete crushing before stirrup yielding, and the specimens exhibited a shear-compression failure mode. However, the test results showed that the ACI 318 code estimated the shear force conservatively. The shear force ratio (*V*_test_/*V*_ACI_) was measured to be 2.01 and 1.57 for specimens 200-C-00 and 100-C-00, respectively. The equations provided in the code gave more conservative results for members with larger stirrup spacings. 

In general, for CFRP-strengthened members, the test results showed that the contribution of the horizontal CFRP strip to the shear and deflection in members with a bidirectional layout was minimal. As shown in Figure 6a, in the case of bidirectionally strengthened specimens of the first series, which were designed with the same total amount of CFRP material as the unidirectionally strengthened members but distributed equally in both vertical and horizontal directions, both the measured maximum shear and deflection were lower than those of the members with the unidirectional CFRP layout. For illustration, specimen 200-B-1F had a total quantity of CFRP strips equal to specimen 200-U-1F but distributed equally in both directions, and the measured maximum shear (*V*_test_) for this specimen was 379 kN, while for the unidirectionally strengthened members, it was 473 kN. For specimens 200-U-1F(R) and 200-B-1F(R), the measured maximum shear forces were 519 and 419 kN, respectively. This shows that the contribution of the horizontal CFRP material to the shear was negligible. Similar trends were observed in the test results of the other specimens of the first series as well. The test results show that despite pre-cracking at the service load level, the maximum shear capacity of the repaired members was slightly increased compared to the members that were strengthened without pre-cracking. For example, specimens 200-U-1F(R) and 100-U-1F(R) had maximum shear values of 519 kN and 581 kN, respectively, which were nearly 9.5% higher than those of members 200-U-1F and 100-U-1F with similar stirrup and CFRP arrangements but without pre-cracking. Similar trends were observed for members with bidirectional CFRP layouts. The shear strengths of specimens 200-B-1F(R) and 100-B-1F(R) were 419 kN and 496 kN, respectively, whereas the specimens 200-B-1F and 100-B-1F showed maximum shear values of 379 kN and 471 kN, respectively.

Although the bidirectionally strengthened members of the second series had a higher total quantity of CFRP material with equal vertical strips when compared with the members with unidirectional layout, in the presence of additional horizontal CFRP strips, the results (Figure 6b) show that the measured maximum shear and deflection were almost the same as those of the members with a unidirectional CFRP layout. For example, the measured shear values for specimens 200-U-1S and 200-B-1S were 304 and 310 kN, respectively. For specimens 200-U-1U, 200-B-1U, 200-U-1F, and 200-B-1F, the measured shear values were 332 kN, 321 kN, 400 kN, and 414 kN, respectively. The test results show that the number of CFRP layers as well as their layout affect the maximum shear. For instance, specimen 200-U-2F had double layers of CFRP strips and therefore showed a higher shear strength of 542 kN compared to specimen 200-B-1F(2) with a measured shear of 429 kN. On the other hand, the shear strength increased when the CFRP layout was changed from side bonded to U-wrapped to fully wrapped. The trend was slightly higher for members with a bidirectional CFRP layout. 

**Figure 7:**
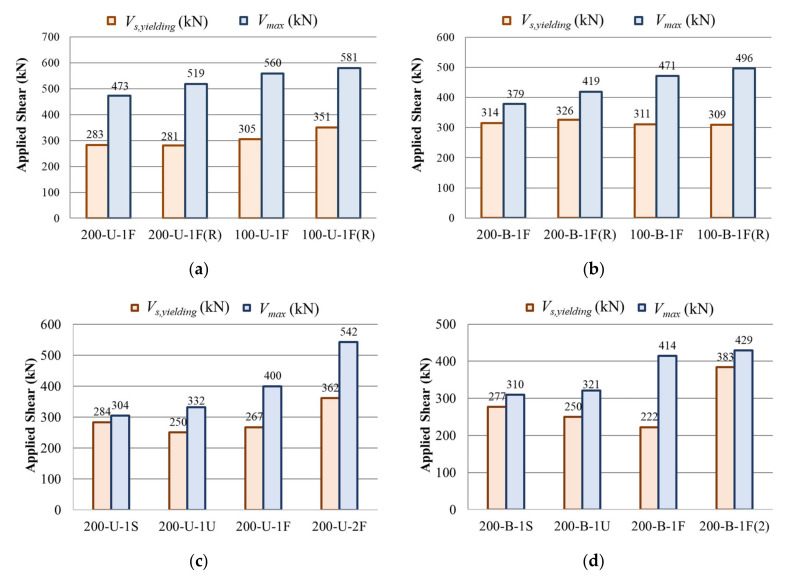
Measured shear force corresponding to stirrup yielding and maximum shear: (**a**) first series: uni-directional layout; (**b**) first series: bi-directional layout; (**c**) second series: uni-directional layout; (**d**) second series: bi-directional layout.

Figure 6 compares the measured shear force corresponding to the stirrup yielding and maximum shear of the specimens in the two series. The specimens were grouped according to CFRP layout. Figure 7a,b show the results of specimens from the first series, while Figure 7c,d present the results of the second series.

### 3.3. Shear Strength Contribution of Materials

**Figure 8:**
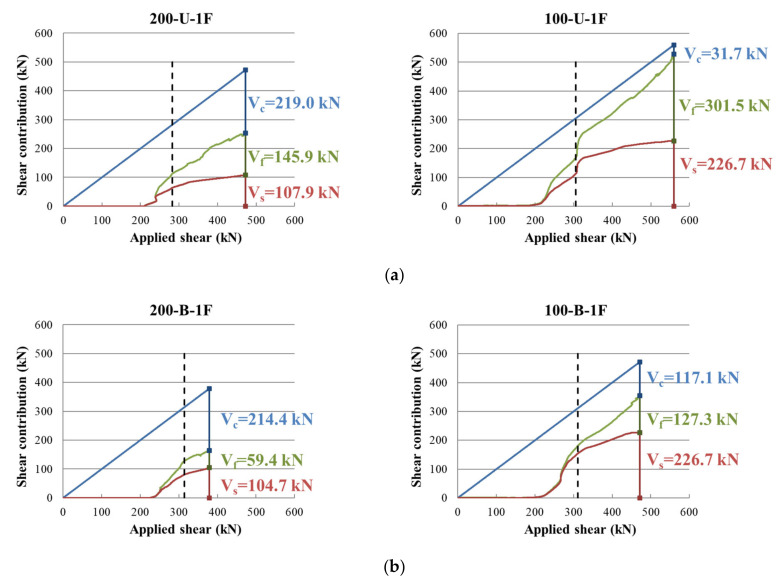

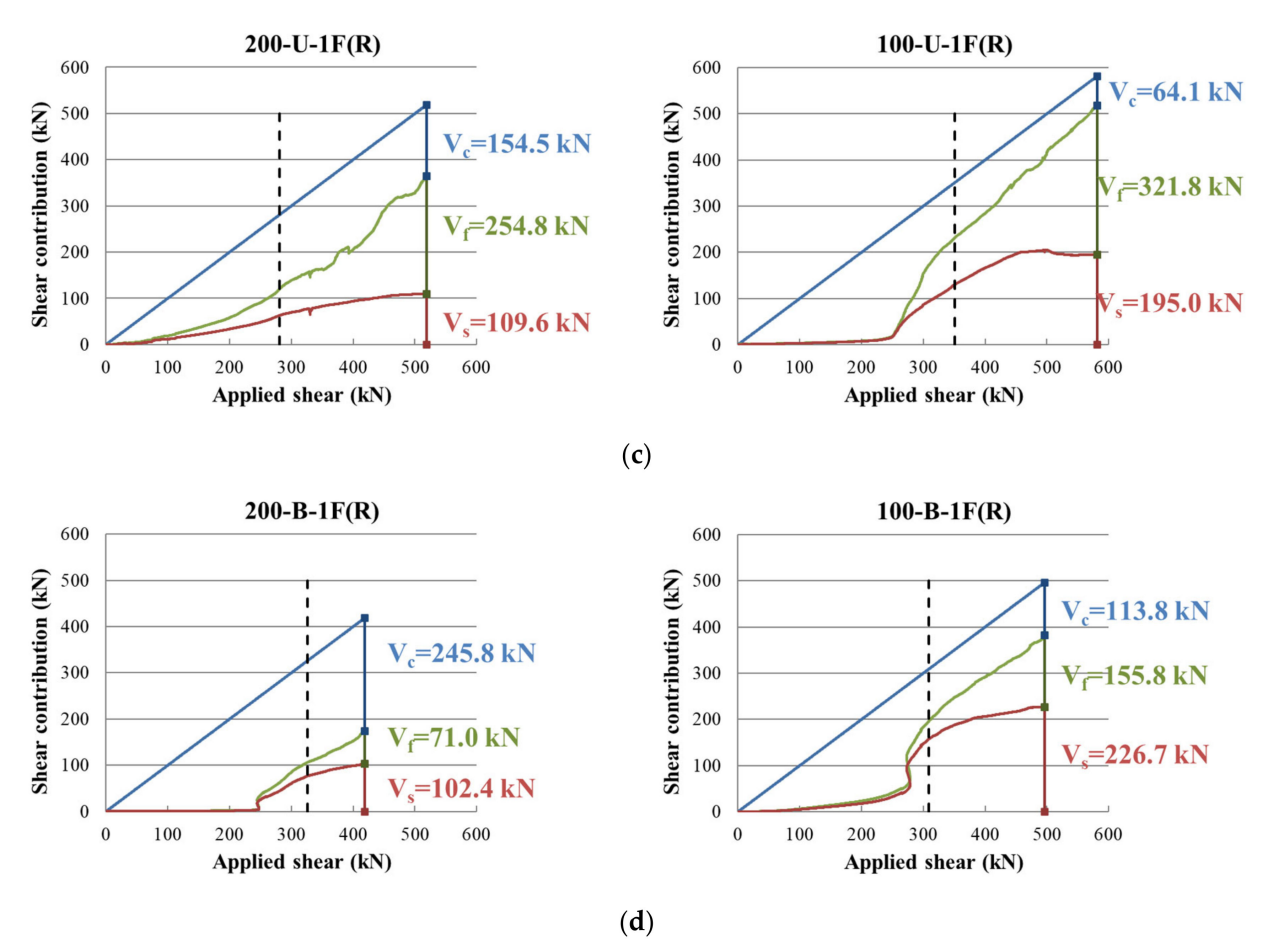
Measured shear contribution of concrete, stirrups and CFRP strips in first series of specimens: (**a**) 200-U-1F vs. 100-U-1F; (**b**) 200-B-1F vs. 100-B-1F; (**c**) 200-U-1F(R) vs. 100-U-1F(R); (**d**) 200-B-1F(R) vs. 100-B-1F(R).

**Figure 9:**
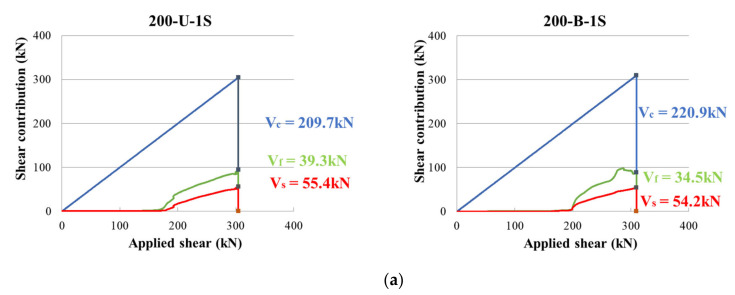

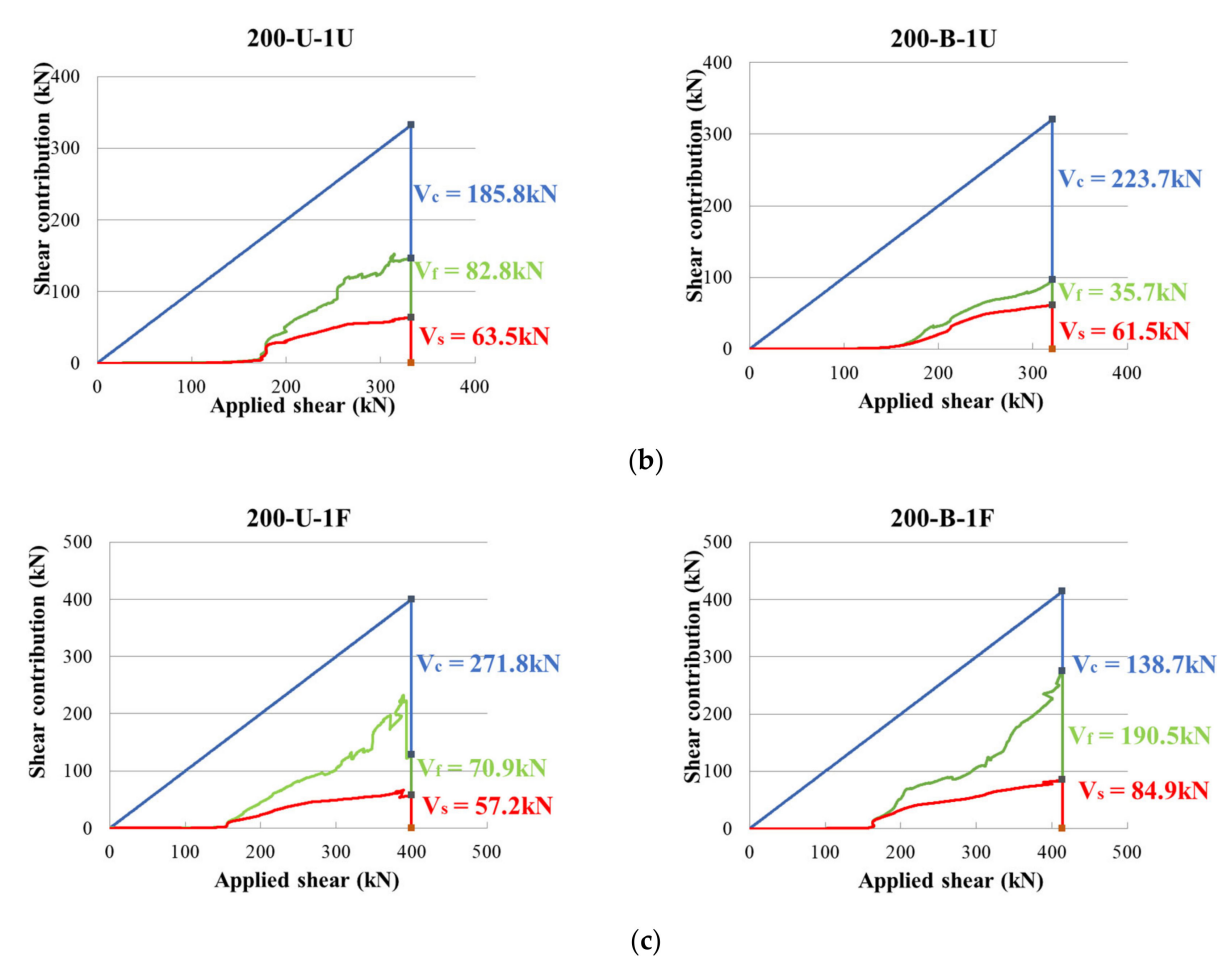
Measured shear contribution of concrete, stirrups and CFRP strips in second series of specimens: (**a**) 200-U-1S vs. 200-B-1S; (**b**) 200-U-1U vs. 200-B-1U; (**c**) 200-U-1F vs. 200-B-1F.

The experimentally obtained shear strength contribution of each material (concrete, stirrups, CFRP, etc.) in two test series is compared in Figure 8 and Figure 9. The *x*-axis and *y*-axis represent the applied shear force and shear contribution of the materials, respectively. In these figures, the dashed vertical line represents the initiation of stirrup yielding. The lower curve represents the shear contribution of the stirrups (Vs), and the middle curve shows the shear contribution of the CFRP strips (Vf). The two material contributions to shear were reverse calculated using the measured strain in stirrups and CFRP strips. Finally, the concrete shear contribution (Vc) was calculated by subtracting the shear contribution of the stirrups and CFRP strips from the applied load. Figure 8 compares the results of the specimens in the first series with identical stirrup layouts and configurations, but different stirrup spacings. Figure 9 shows the results of the second series of specimens with the same stirrup spacing and CFRP layouts, categorized according to the CFRP layout, that is, unidirectional, bidirectional, etc. As shown in the figure, the shear resistance contribution of the stirrups and CFRP strips started after concrete cracking. Most of the stirrups yielded before reaching the maximum load. The increase in the rate of the shear contribution of the CFRP strips became steeper with the initiation of the stirrup yielding. The concrete shear contribution tended to decrease as the stirrup spacing decreased. However, the CFRP shear contribution tended to increase as the stirrup spacing decreased. 

The numerical values of the shear contribution of each material are presented in Table 4. These are presented as ratios of the measured values to the ACI318-19 code prediction (*V*_c,test_/*V*_c,ACI_), (*V*_s,test_/*V*_s,ACI_), (*V*_f,test_/*V*_f,ACI_), etc., where *V*_c_, *V*_s_, and *V*_f_ are the shear contributions of concrete, stirrups, and CFRP strips, respectively. Generally, the ACI318 code underestimated the contribution of all the materials. In particular, the stirrup and CFRP contributions to shear were considerably underestimated in many cases. The test results show that for members designed for equal or higher CFRP shear contribution than that of stirrups (*V*_f,ACI_/*V*_s,ACI_ equal to or greater than 1), the contribution of CFRP strips (*V*_f,test_/*V*_s,test_) was very high. For example, specimens 200-U-1F and 100-U-1F were designed to have an 18% higher CFRP shear contribution than the stirrups. The test results showed that the actual shear contribution of the CFRP was 35% and 33% higher than that of the stirrup. This increasing trend of CFRP shear contribution was higher in pre-cracked and repaired members, that is, specimens 200-U-1F(R) and 100-U-1F(R), than in members without pre-cracking. For members designed with *V*_f,ACI_/*V*_s_, and ACI less than 1, the measured *V*_f,test_/*V*_s,test_ increased slightly. In general, the ratios of the test results to the design values increased with a narrower stirrup spacing. The average increase was 1.3 times in the case of 200 mm stirrup spacing and 2.3 times in 100 mm stirrup spacing. These results indicate that the strengthening effect of the CFRP strips can vary depending on the amount of transverse reinforcement. The strengthening effect of CFRP was greater when more stirrups were used.

### 3.4. Stirrup Strain

**Figure 10:**
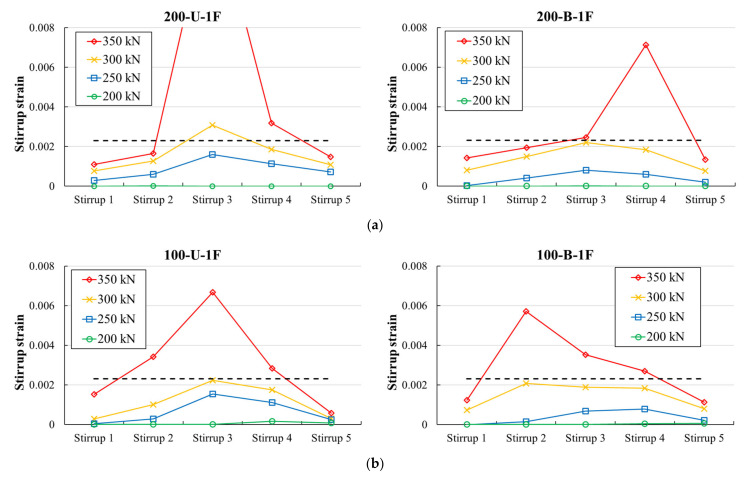

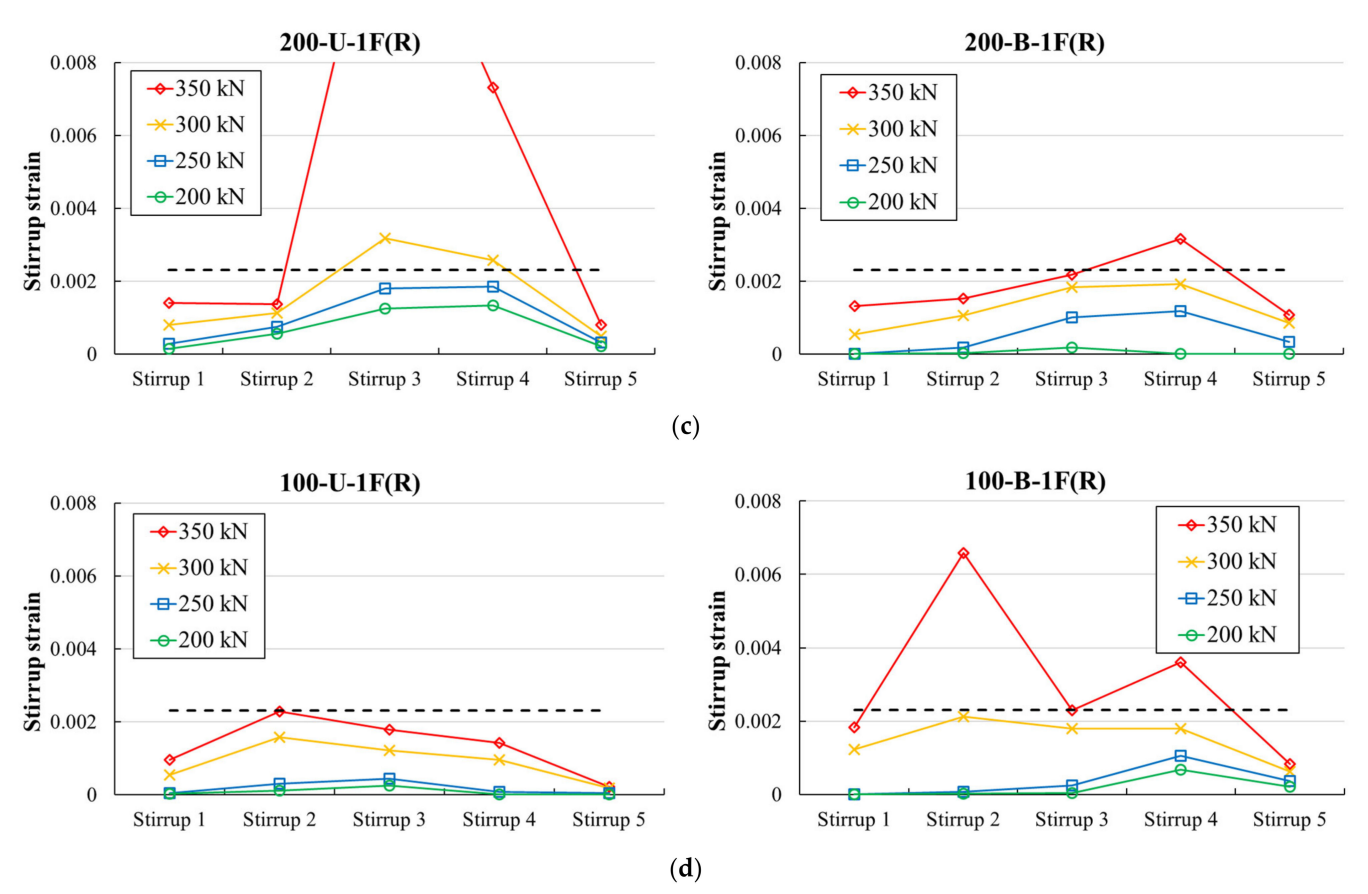
Measured stirrup strains of first series of specimens: (**a**) 200 mm spacing; (**b**) 100 mm spacing; (**c**) 200 mm spacing—repaired; (**d**) 100 mm spacing—repaired.

**Figure 11:**
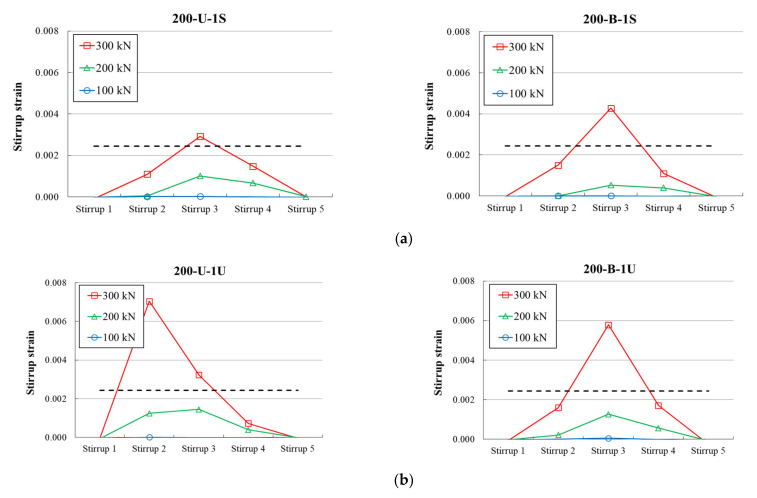

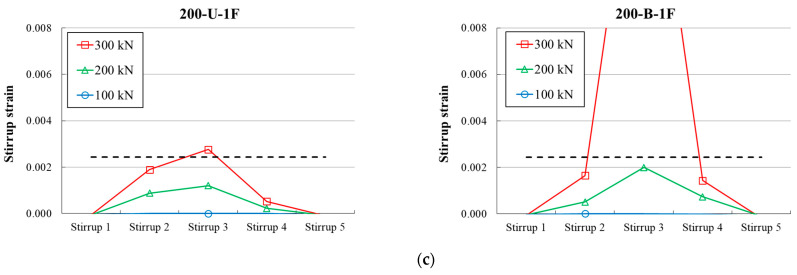
Measured stirrup strains of second series of specimens: (**a**) 200 mm spacing; (**b**) 100 mm spacing; (**c**) 100 mm spacing—repaired.

Figure 10 and Figure 11 present the measured stirrup strains of the test specimens according to the stirrup location. The x- and y-axes represent the location of the stirrup and recorded strains, respectively. Stirrup 1 is a gauge attached to a stirrup near the left reaction point of the specimen, and Stirrup 5 is a gauge attached to a stirrup near the loading point of the specimen. The measured strains corresponding to different loading levels are presented as different colored lines. The dashed horizontal line represents the calculated yield strain based on the measured material properties. In the case of specimens in the first series, at a loading level of 350 kN, the measured strain in the stirrups of all specimens reached the yield strain. A comparison of the results demonstrates that the measured maximum stirrup strain in all members with a unidirectional CFRP strip layout was higher than that of members with a bidirectional layout, except for specimen 100-U-1F(R). For illustration, the measured maximum stirrup strain corresponding to a chosen load level of 350 kN in specimens 200-U-1F, 200-U-1F (R), and 100-U-1F were 0.0146, 0.0139, and 0.0067, respectively. For specimens 200-B-1F, 200-B-1F(R), and 100-U-1F specimens, the strains were 0.0071, 0.0057, and 0.0032, respectively. 

Similarly, at a fixed load level of 300 kN, at least one or more stirrups in the specimens of the second series reached the yield strain. The results showed that the configuration of the CFRP strips affected the stirrup strain. Except for specimen 200-U-1F, the measured maximum stirrup strain at a fixed load level was larger in all specimens with U-wrapped and fully wrapped configurations than in specimens with side-bonded configurations. For instance, the measured maximum strain in specimen 200-B-1F was 0.0193, which was much higher than the strain 0.0043 of specimen 200-B-1S with a side-bonded configuration. Similarly, the strain in specimen 200-U-1U was measured to be 0.0070, and in the case of specimen 200-U-1S, the strain was 0.0029. The measured strain of the specimen 200-U-1F was recorded to be 0.0019.

## 4. Discussion

As was intended in the design, none of the specimens exhibited flexural yielding. All the CFRP-strengthened specimens failed due to strip fracture, except those with side-bonded configurations. Unlike reported previously [14,24,28,29], the contribution of the horizontal CFRP strips to shear and deflection in members with a bidirectional layout was negligible in general. The bidirectionally strengthened specimens of the first series were designed with the same total quantity of CFRP material as the unidirectionally strengthened members but were distributed equally in the vertical and horizontal directions; both the measured maximum shear and deflection were lower than those of members with the unidirectional CFRP layout. Even with a higher total amount of CFRP material, the measured maximum shear and deflection of the bidirectionally strengthened members of the second series were almost the same as those with a unidirectional CFRP layout, which is contrary to the results reported by Yungon et al. [14], Nawaf [25], and Kim et al. [26]. Yungon et al. [14] and Nawaf [25] reported that in members with bidirectional CFRP strips, the shear strength was 40% higher than in the case of members with a unidirectional configuration. Despite the pre-cracking, the maximum shear capacity of the repaired members was slightly higher than that of the members strengthened without pre-cracking. For instance, specimen 200-U-1F(R) had a maximum shear of 519 kN, which was nearly 9.5% higher than that of members of specimen 200-U-1F without pre-cracking. Similar trends were also observed for members with bidirectional CFRP layouts. The pre-cracked and repaired specimens had a higher shear contribution of the CFRP strips than the pre-strengthened specimens. The location of maximum strain in CFRP caused by concrete cracks (pre-cracked) maximized the shear contribution of the CFRP materials. Although an identical amount of CFRP material was used, the higher shear capacity of the steel reinforcement (narrow stirrup spacing) resulted in a higher shear contribution of the CFRP strips. This implies that the strengthening effect of CFRP materials can be maximized when the shear contribution of the steel reinforcement increases. The shear contribution of the CFRP strips increased after stirrup yielding. Similar observations were reported by Yungon et al. [14]. The shear contribution of concrete decreased with decreasing stirrup spacing. In members designed to have a CFRP shear contribution equal to or higher than that of stirrups, the contribution of the CFRP strips was very high. This increase in the CFRP shear contribution was higher for the pre-cracked and repaired members. The bidirectional CFRP layout allowed well-distributed stirrup strain compared to unidirectional CFRP layout at the same load level. This implies that the use of a bidirectional CFRP layout can increase the efficiency of transverse reinforcement. A comparison of the results demonstrates that the measured maximum stirrup strain at a fixed load level was larger in all specimens with U-wrapped and fully wrapped configurations than in specimens with side-bonded configurations. The shear capacity of specimens with a fully wrapped unidirectional CFRP layout was higher than that for the specimens with a U-wrapped (or side-bonded) bidirectional CFRP layout. The use of horizontal CFRP strips could not completely prevent the delamination failure of the vertical CFRP strips. This indicates that CFRP anchors cannot be effectively replaced by horizontal CFRP strips.

## 5. Conclusions

In this study, the effectiveness of bidirectional CFRP strengthening for rectangular RC beams was experimentally investigated. The studied parameters were the spacing of stirrups, the amount, layout, and configuration of the CFRP strips, and the effect of pre-cracking. The following important conclusions can be drawn from the results of this study:

1. All the specimens showed ultimate failure due to strip fracture, except the specimens with side-bonded configuration in which bond delamination governed the behavior. Even with higher total amount of CFRP material, the contribution of the horizontal CFRP strips to shear and deflection in members with a bidirectional layout was negligible.
2. Despite pre-cracking, the maximum shear capacity of the repaired members was slightly higher than that of the members strengthened without pre-cracking. The repaired specimens had a higher shear contribution of the CFRP strips than the pre-strengthened specimens.
3. With identical amount of CFRP material, the members with higher amount of transverse reinforcement showed a higher shear contribution of the CFRP strips. This implies that the strengthening effect of CFRP materials can be maximized when the spacing of stirrups is reduced.
4. The use of a bidirectional CFRP layout increased the efficiency of transverse reinforcement. A comparison of the results demonstrates that the measured stirrup strain at a fixed load level was well-distributed in members with bidirectional CFRP layout in members with unidirectional layout.
5. The horizontal CFRP strips could not completely prevent the delamination failure of the vertical CFRP strips in members with side-bounded configuration. This indicates that CFRP anchors cannot be effectively replaced by horizontal CFRP strips in case of bidirectional layout.

## Figures and Tables

**Table 1:** Test specimen details.

Specimen Index	Cross-Sectional Details		*f*_c_’ (MPa)	CFRP Strip					Stirrup Spacing, s (mm)
	*b* (mm)	*h* (mm)		*f_f_* (MPa)	*w_f,v_* (mm)	*s_f,v_* (mm)	*w_f,h_* (mm)	*s_f,h_* (mm)	
200-C-00	300	500	36.8	N/A					200
200-U-1F	300	500	36.8	4600	100	200	N/A		200
200-U-1F(R)	300	500	36.8	4600	100	200			200
200-B-1F	300	500	36.8	4600	50	200	50	200	200
200-B-1F(R)	300	500	36.8	4600	50	200	50	200	200
100-C-00	300	500	36.8	N/A					100
100-U-1F	300	500	36.8	4600	100	200	N/A		100
100-U-1F(R)	300	500	36.8	4600	100	200			100
100-B-1F	300	500	36.8	4600	50	200	50	100	100
100-B-1F(R)	300	500	36.8	4600	50	200	50	100	100
200-U-1S	300	500	31.8	4600	100	200	N/A	200	200
200-U-1U	300	500	31.8	4600	100	200		200	200
200-U-1F	300	500	31.8	4600	100	200		200	200
200-U-2F	300	500	31.8	4600	100	200		200	200
200-B-1S	300	500	31.8	4600	100	200	100	200	200
200-B-1U	300	500	31.8	4600	100	200	100	200	200
200-B-1F	300	500	31.8	4600	100	200	100	200	200
200-B-1F (2)	300	500	31.8	4600	100	200	100	200	200

(Note *b*: width of RC beam, *h*: height of RC beam, *f_c_’*: concrete strength, *f_f_*: ultimate strength of CFRP material, *w_f,v_*: vertical CFRP strip width, *s_f,v_*: vertical CFRP strip spacing, *w_f,h_*: horizontal CFRP strip width, *s_f,h_*: horizonal CFRP strip spacing).

**Table 2:** Material properties.

Test Parameters		Transverse Reinforcement, D6	Longitudinal Reinforcement, D25
First Test Series	*f*_y_ (MPa)	340	660
	*ε*_y_	0.0023	0.0034
	*E* (MPa)	148,000	195,000
Second Test Series	*f*_y_ (MPa)	389	400
	*ε*_y_	0.0024	0.0021
	*E* (MPa)	159,000	195,000

**Table 3:** Test results.

Specimen	*V*_ACI_ (kN)	*V*_test_ (kN)	*V*_test_/*V*_ACI_	*ε*_s,max_	*ε*_f,max_	Failure Mode
200-C-00	179	360	2.01	0.0147	-	Shear-compression
200-U-1F	235	473	2.01	0.0133	0.0075	Strip fracture
200-U-1F(R)	235	519	2.21	0.0150	0.0101	Strip fracture
200-B-1F	207	379	1.83	0.0137	0.0081	Strip fracture
200-B-1F(R)	207	419	2.02	0.0109	0.0079	Strip fracture
100-C-00	226	356	1.57	0.0131	-	Shear-compression
100-U-1F	282	560	1.99	0.0163	0.0127	Strip fracture
100-U-1F(R)	282	581	2.06	0.0163	0.0126	Strip fracture
100-B-1F	254	471	1.85	0.0143	0.0102	Strip fracture
100-B-1F(R)	254	496	1.95	0.0157	0.0104	Strip fracture
200-U-1S	212	304	1.43	0.0046	0.0062	Strip delimitation
200-U-1U	213	332	1.56	0.0033	0.0073	Strip fracture
200-U-1F	219	400	1.83	0.0040	0.0170	Strip fracture
200-U-2F	269	542	2.01	0.0031	0.0103	Strip fracture
200-B-1S	212	310	1.46	0.0075	0.0047	Strip delimitation
200-B-1U	213	321	1.50	0.0094	0.0024	Strip fracture
200-B-1F	219	414	1.89	0.0142	0.0103	Strip fracture
200-B-1F (2)	219	429	1.96	0.0113	0.0095	Strip fracture

Note: *V*_test_, *V*_ACI_ = Measured and calculated shear strength using ACI318-19, ***ε*_s,max_*, ε*_f,max_** = measured maximum strain in stirrups and CFRP strips.

**Table 4:** Shear contribution of each material.

Specimen	*V*_c,test_/*V*_c,ACI_	*V*_s,test_/*V*_s,ACI_	*V*_f,test_/*V*_f,ACI_	*V*_f,ACI_/*V*_s,ACI_	*V*_f,test_/*V*_s,test_
200-C-00	1.96	2.13	-	-	-
200-U-1F	1.66	2.29	2.63	1.18	1.35
200-U-1F(R)	1.17	2.33	4.60	1.18	2.32
200-B-1F	1.62	2.22	2.14	0.59	0.57
200-B-1F(R)	1.86	2.17	2.56	0.59	0.69
100-C-00	1.24	2.04	-	-	-
100-U-1F	0.24	2.41	5.44	0.59	1.33
100-U-1F(R)	0.48	2.07	5.81	0.59	1.65
100-B-1F	0.89	2.41	4.60	0.29	0.56
100-B-1F(R)	0.86	2.41	5.62	0.29	0.69
200-U-1S	1.79	1.08	0.76	1.00	-
200-U-1U	1.58	1.24	1.57	1.03	0.71
200-U-1F	2.32	1.11	1.34	1.03	1.30
200-U-2F	2.42	1.76	1.59	2.06	1.24
200-B-1S	1.88	1.05	0.67	1.00	1.86
200-B-1U	1.91	1.20	0.67	1.03	0.64
200-B-1F	1.18	1.66	3.60	1.03	0.58
200-B-1F (2)	2.60	1.58	0.81	1.03	2.24

Note: *V*_c,test_, *V*_s,test_, *V*_f,test_ = Measured concrete, stirrups and CFRP shear contribution, *V*_c,ACI_, *V*_s,ACI_, *V*_f,ACI_ = concrete, stirrups and CFRP shear contribution calculated through ACI318-19.

## Data Availability

All the research data used in this manuscript will be available whenever requested.
